# Maternal age and oral health–related quality of life in preschool children: the mediating role of dental caries in a cross-sectional study

**DOI:** 10.3389/froh.2026.1863687

**Published:** 2026-08-20

**Authors:** Belén Castillo, Domingo Lancellotti

**Affiliations:** 1 Centro de Salud Familiar El Sauce, Coquimbo, Chile; 2 Departamento de Salud Pública, Universidad Católica del Norte, Coquimbo, Chile

**Keywords:** early childhood caries, health inequalities, maternal age, mediation analysis, oral health–related quality of life

## Abstract

**Introduction:**

Early Childhood Caries (ECC) is a highly prevalent chronic condition that disproportionately affects children in socioeconomically vulnerable settings and negatively impacts oral health–related quality of life (OHRQoL). While maternal age has been identified as a relevant factor, the mechanisms linking maternal age to children's oral health outcomes remain insufficiently understood. This study aimed to examine the relationship between maternal age, dental caries experience, and OHRQoL, and assess the mediating role of dental caries in this relationship.

**Methods:**

A cross-sectional study was conducted in 183 mother–child dyads involving 4- and 5-year-old children attending a primary healthcare center in Coquimbo, Chile. Dental caries experience was assessed using the decayed, missing, and filled teeth (dmft) index, and children's OHRQoL was measured using the Early Childhood Oral Health Impact Scale (ECOHIS). Statistical analyses included non-parametric tests for group comparisons, regression analyses, and mediation analysis using a regression-based approach with natural log-transformed variables.

**Results:**

The mean maternal age at childbirth was 22.6 ± 6.7 years (range: 12–39 years), with mothers distributed across the full maternal age continuum. Caries prevalence was 55.7%, with a mean dmft score of 2.25 ± 2.63, and the mean total ECOHIS score was 18.4. Maternal age was inversely associated with both dental caries experience and children's OHRQoL impairment (both *p* < 0.001). Dental caries experience was strongly associated with poorer children's OHRQoL, explaining 85.5% of the variability in ECOHIS scores (*p* < 0.001). Mediation analysis indicated that dental caries represented an important pathway linking maternal age to children's OHRQoL, accounting for 57.8% of the total association. The negative indirect effect (*p* < 0.001) was consistent with a protective pathway whereby increasing maternal age was associated with better children's OHRQoL through reduced dental caries experience.

**Conclusion:**

The mediation analysis suggested that dental caries constituted an important component of the association between maternal age and children's OHRQoL. The analysis of maternal age as a continuum provided a more comprehensive understanding of inequalities in early childhood oral health and reinforced its interpretation as a marker of broader social and contextual conditions influencing children's oral health. These findings support life-course and equity-oriented strategies for improving children's oral health and reducing oral health inequalities.

## 1 Introduction

Early childhood caries (ECC) is one of the most prevalent chronic diseases in childhood. According to the 2021 Global Burden of Disease Study, ECC affected approximately 525 million children worldwide—49.0% of the global pediatric population—with an estimated prevalence of 7.55 per 100,000 inhabitants. Although widespread, ECC shows marked variability at global, regional, and national levels, largely driven by sociodemographic inequalities (1–5). Higher ECC burden has been consistently associated with lower economic development, low socioeconomic status, high sugar intake, low maternal educational level, prolonged breastfeeding, and nocturnal feeding, whereas appropriate oral hygiene practices and caregiver-assisted toothbrushing offer protective effects (6–9).

ECC substantially compromises children's oral health–related quality of life (OHRQoL), with the magnitude of its impact increasing alongside disease severity. Low socioeconomic position and lower maternal educational attainment have also been associated with poorer OHRQoL, while interventions aimed at reducing ECC have demonstrated improvements in quality of life for both children and their families (10–18).

In Chile, recent studies have reported a decline in ECC prevalence among preschool children, largely attributed to sustained public oral health policies and long-standing preventive programs (19–21). Despite these improvements, the burden of disease remains high. In 2021, an estimated 992,000 Chilean children were affected by ECC, corresponding to a prevalence of 7.95 per 100,000 inhabitants—exceeding the global average (1). Within the country, ECC disproportionately affects children from lower socioeconomic strata (22, 23), those whose mothers have lower educational attainment (24), and those living in rural areas, who experience an increased likelihood of poor oral health outcomes (21, 25–30). National studies addressing OHRQoL in preschool children have also reported an inverse association between OHRQoL and disease severity (31–33). Several of these studies have examined maternal age as a variable of interest, most reporting inverse but weak associations with either ECC or OHRQoL. Nonetheless, evidence indicates that younger maternal age may exert a meaningful influence on children's oral health, with adolescent motherhood identified as a particularly important risk factor (11, 34–38). In Chile, however, the influence of maternal age across the continuum, from adolescent motherhood onward, on children's OHRQoL—and the contribution of dental caries to this relationship—remains insufficiently explored.

Based on the available evidence, dental caries may represent an important intermediate mechanism linking maternal age to children's OHRQoL. Accordingly, differences in children's dental caries experience may explain part of this association, providing the rationale for evaluating dental caries as a potential mediator. The present study examines the association between maternal age and oral health–related quality of life in preschool children, and evaluates the mediating role of dental caries experience across the full maternal age continuum in an urban, middle–low socioeconomic population.

## 2 Materials and methods

### 2.1 Study design and setting

This cross-sectional study was conducted in 2023 at a Family Health Center located in a middle-to-low income urban area of Coquimbo, north-central Chile. The study population comprised preschool children enrolled in the state dental program “CERO” (Spanish acronym for Risk-Based Dental Care Program), which provides preventive and therapeutic oral healthcare for children aged 3–5 years. Mothers attending their child's first dental appointment were invited to participate. Data collection was conducted on-site following standardized procedures commonly used in oral health epidemiological studies.

### 2.2 Ethical considerations

The study protocol was approved by the Scientific Ethics Committee of the Faculty of Medicine, Universidad Católica del Norte (Res. CECFAMED-UCN REV. No. 50/2023). All participating mothers were aged ≥18 years and provided written informed consent prior to participation. All procedures were conducted in accordance with the principles of the Declaration of Helsinki.

### 2.3 Participants and sample size

Eligible participants were mother–child dyads involving children aged 4 or 5 years who had not yet received dental care through the CERO program. This age range was selected because the first dental intervention under the program begins at 4 years of age. Children with systemic conditions affecting oral health or special healthcare needs that could preclude clinical examination were excluded.

The target population consisted of 192 eligible children enrolled at the Family Health Center in June 2023. Prior to data collection, a sample size calculation was performed considering this finite population of 192 children, a 95% confidence level, a precision of 5%, and an expected caries prevalence of 50%, which was assumed because it yields the largest sample size estimate among all possible prevalence values. The sample size was estimated at 128 mother–child dyads. However, a total of 183 mother–child dyads were ultimately included, representing 95.3% of the target population. Thus, only nine eligible children were not included in the study.

### 2.4 Data collection procedures

All oral examinations were performed by the same clinician using standardized diagnostic criteria. The use of a single examiner was intended to enhance consistency across clinical assessments. No formal calibration exercise or reproducibility assessment was conducted. Questionnaires were administered by trained personnel under the supervision of the clinician. Face-to-face interviews were conducted in a private room to ensure confidentiality and minimize response bias.

### 2.5 Dental caries experience

Dental caries experience was assessed using the decayed, missing, and filled teeth index (dmft), following the diagnostic criteria established by the World Health Organization (WHO) for oral health surveys (39). Scores range from 0 upward, with higher values indicating greater disease burden and treatment needs. Caries prevalence was calculated as the proportion of children with dmft >0.

### 2.6 Oral health-related quality of life

Children's OHRQoL was assessed using the Early Childhood Oral Health Impact Scale (ECOHIS), an internationally validated instrument for preschool children (40, 41) that has also been validated in the Chilean population (31). The ECOHIS comprises 13 items distributed across two dimensions: the Child Impact dimension, which includes four domains (oral symptoms, functional limitations, psychological impacts, and self-image/social interaction), and the Family Impact dimension, which includes two domains (parental distress and family function). Responses are scored on a five-point Likert scale ranging from 0 (“never”) to 4 (“very often”). Total scores range from 0 to 52, with higher scores indicating poorer children's OHRQoL.

For descriptive purposes and to facilitate the interpretation of item-level responses, ECOHIS response categories were grouped into broader categories, following an approach previously used in some studies (40, 42, 43). Responses of “Never” and “Hardly ever” were combined into a single category representing a positive connotation, whereas “Occasionally”, “Often”, and “Very often” were combined into a category reflecting a negative connotation.

### 2.7 Sociodemographic variables

Information was collected for the child (age and sex) and the mother (age at childbirth and educational attainment). Maternal age at childbirth was analysed as a continuous variable in regression and mediation analyses. For descriptive purposes, maternal age was additionally categorized into age groups to facilitate presentation of prevalence estimates and disease gradients across the maternal age continuum. Maternal educational attainment at the time of interview was classified as secondary, technical–professional, or university education.

### 2.8 Statistical analysis

Descriptive statistics were calculated for all variables. Continuous variables were summarized using means and standard deviations (SD), medians and interquartile ranges (IQR), or both, as appropriate, whereas categorical variables were summarized using frequencies and percentages.

Prior to group comparisons, normality was assessed using the Shapiro–Wilk test, whereas homogeneity of variances was assessed using both Levene and Bartlett tests. Because these assumptions were not satisfied, non-parametric statistical methods were used. The Mann–Whitney *U*-test was applied for comparisons between two groups, whereas the Kruskal–Wallis test followed by Dunn's *post hoc* test with Bonferroni correction was used for comparisons among three or more groups. Chi-square tests were used for categorical variables.

For regression analyses examining the relationships among maternal age, dental caries experience, and children's OHRQoL, alternative functional forms were evaluated according to statistical fit and conceptual interpretability. Model adequacy was assessed through diagnostic procedures evaluating heteroscedasticity using the Breusch–Pagan and White tests and model specification using the RESET test. Because dental caries experience and ECOHIS scores contained zero values, logarithmic transformations were applied after adding a constant of 1 [ln(dmft+1) and ln(ECOHIS+1)]. The addition of a constant prior to logarithmic transformation is a commonly used approach that allowed retention of all observations (44). Exponential regression models were selected for the maternal age–dental caries experience and maternal age–children's OHRQoL relationships, whereas a logarithmic regression model was selected for the dental caries experience–children's OHRQoL relationship.

Potential effect modification by child sex and age was explored through interaction models and Wald tests. Because no statistically significant interactions were observed, all subsequent analyses were conducted using the pooled sample (see Supplementary Table S1).

### 2.9 Mediation analysis

Mediation analysis was conducted to examine whether dental caries experience mediated the association between maternal age and children's OHRQoL using a regression-based framework (45). Maternal age was specified as the exposure variable (X), dental caries experience as the mediator (M), and children's OHRQoL as the outcome (Y). All three variables were analysed as continuous measures. Maternal age was retained in its original scale, whereas ln(dmft+1) and ln(ECOHIS+1) were used in mediation models.

The mediation model estimated: (i) the association between maternal age and ln(dmft+1) (path *a*), (ii) the association between ln(dmft+1) and ln(ECOHIS+1) after adjustment for maternal age (path *b*), (iii) the total association between maternal age and ln(ECOHIS+1) (path *c*), and (iv) the direct association between maternal age and ln(ECOHIS+1) after accounting for dental caries experience (path *c*′). Accordingly, the estimates reported for paths *a*, *b*, *c*, and *c*′ are the corresponding regression coefficients from these linear regression models. The indirect effect was calculated as the product *a*x*b*, and the proportion mediated was estimated as (*a*x*b*)/*c.* The indirect effect was assessed using the Sobel test (46) and non-parametric bootstrap confidence intervals.

### 2.10 General statistical considerations

For all statistical analyses, statistical significance was established at *p* < 0.05. When applicable, results are presented with 95% confidence intervals (95% CI). All analyses were performed using R (47) and XLSTAT (48).

## 3 Results

### 3.1 Participant characteristics and oral health outcomes

Table 1 presents the sociodemographic characteristics of the children and their mothers, together with the main oral health outcomes. Of the 183 preschool children included in the study, 52.5% were girls and 51.4% were 5 years old. No statistically significant differences were observed by sex or age (*p* ≥ 0.554). The mean maternal age at childbirth was 22.6 ± 6.7 years, with a median of 21 years (IQR: 17–27; range: 12–39 years); 26.2% of mothers were younger than 18 years, and 13.1% were younger than 15 years, with no statistically significant differences in the distribution of mothers across maternal age groups (*p* = 0.236). Most mothers had secondary (45.9%) or technical–professional (47.5%) education at the time of the interview, with 51.2% and 23.0% of these groups reporting incomplete studies, respectively. Only 6.6% reported university education (*p* < 0.001), of whom 33.3% had not completed that level. Mothers with secondary education had a significantly lower mean age at childbirth, being on average 5.5 years younger than mothers with technical–professional education and 7.2 years younger than those with university education (*p* < 0.001). The prevalence of caries experience among children was 55.7%, with a mean dmft index of 2.25. No significant differences were observed according to sex or age in either prevalence (*p* ≥ 0.221) or mean dmft index (*p* ≥ 0.140), although children aged 5 years showed consistently higher values than children aged 4 years. Caries experience exceeded 90% among children of adolescent and very young mothers (≤20 years) and declined sharply with increasing maternal age, reaching 3.8% among children of mothers aged ≥31 years (*p* < 0.001). Mean dmft values followed a similar gradient, decreasing from 4.8 to 0.04 (*p* < 0.001).

**Table 1:** Sociodemographic characteristics of the study population and children's oral health outcomes.

Sociodemographic variable	*n* (%)	*p*-value^a^	Age: mean (SD)	*p*-value^b^
Child characteristics	*n* (%)	*p*-value^a^		
Sex				
Girls	96 (52.5)	0.554		
Boys	87 (47.5)			
Age (years)				
4	89 (48.6)	0.767		
5	94 (51.4)			
Maternal characteristics	*n* (%)	*p*-value^a^	Age: mean (SD)	*p*-value^b^
Age at childbirth (years)				
≤14	24 (13.1)	0.236		
15–17	24 (13.1)			
18–20	37 (20.2)			
21–25	36 (19.7)			
26–30	36 (19.7)			
≥31	26 (14.2)			
Education level (at interview)				
Secondary education	84 (45.9)	**<0.001**	19.5 (5.4)^A^	**<0.001**
Technical–professional education	87 (47.5)		25.0 (6.5)^B^	
University education	12 (6.6)		26.7 (7.0)^B^	
Children's oral health outcomes				
Child characteristics	Caries experience prevalence (%)	*p*-value^a^	dmft: mean (SD); median (IQR)	*p*-value^b^
Sex				
Girls	57.3	0.768	2.2 (2.6); 2 (0–3.3)	0.816
Boys	54.0		2.3 (2.9); 2 (0–4)	
Age (years)				
4	50.6	0.221	2.0 (2.7); 1 (0–3)	0.140
5	60.6		2.5 (2.8); 2 (0–4)	
Maternal characteristics	Caries experience prevalence (%)	*p*-value^a^	dmft: mean (SD); median (IQR)	*p*-value^b^
Age at childbirth (years)				
≤14	91.7	**<0.001**	4.8 (3.6); 4 (2–7.3)^A^	**<0.001**
15–17	95.8		4.7 (2.6); 4 (3–5.2)^A^	
18–20	91.9		3.3 (2.2); 3 (2–5)^A^	
21–25	50.0		1.5 (1.9); 1 (0–3)^B^	
26–30	11.1		0.2 (0.6); 0 (0–0)^B^	
≥31	3.8		0.0 (0.2); 0 (0–0)^B^	

a *p*-values for caries prevalence were obtained using the Chi-square test.

b *p*-values for dmft comparisons were obtained using the MannWhitney U test (for sex and children's age) and the KruskalWallis test (for maternal age at childbirth). Statistically significant differences (*p* < 0.05) are highlighted in bold. Different uppercase letters denote statistically significant differences between groups (*p* < 0.05) according to Dunn's post hoc test with Bonferroni correction.

### 3.2 Children's oral health-related quality of life

Table 2 presents maternal responses to the Early Childhood Oral Health Impact Scale (ECOHIS). In the Child impact dimension, responses with a positive connotation predominated across most items, with statistically significant differences observed for “Had difficulty pronouncing any words”, “Been irritable or frustrated”, “Avoided smiling or laughing”, and “Avoided talking”. The exception was the item “Had pain in the teeth, mouth, or jaws”, which showed a minimal difference favoring negative connotation, with no statistically significant differences. In the Family impact dimension, responses with a negative connotation predominated for all items; however, none of these differences reached statistical significance. Across all ECOHIS items, 56.7% of responses were positively connoted (*p* < 0.001), indicating that most responses were concentrated in the low-frequency impact categories.

**Table 2:** Distribution of ECOHIS responses by item within domains among preschool children.

Impacts	Never or hardly ever *n* (%)	Occasionally, often or very often *n* (%)	*p*-value
Child impact dimension			
Oral symptoms domain			
1. Had pain in the teeth, mouth, or jaws?	90 (49.2)	93 (50.8)	0.882
Functional limitations domain			
2. Had difficulty drinking hot or cold beverages?	96 (52.5)	87 (47.5)	0.554
3. Had difficulty eating some foods?	94 (51.6)	88 (48.4)	0.711
4. Had difficulty pronouncing any words?	155 (84.7)	28 (15.3)	**<0**.**001**
5. Missed preschool, day care or school?	97 (53.0)	86 (47.0)	0.460
Psychological impacts domain			
6. Had trouble sleeping?	102 (55.7)	81 (44.3)	0.139
7. Been irritable or frustrated?	113 (61.7)	70 (38.3)	**0**.**002**
Self-image/social interaction domain			
8. Avoided smiling or laughing?	136 (74.3)	47 (25.7)	**<0**.**001**
9. Avoided talking?	121 (66.1)	62 (33.9)	**<0**.**001**
Family impact dimension			
Parental distress domain			
10. Been upset?	90 (49.2)	93 (50.8)	0.882
11. Felt guilty?	86 (47.0)	97 (53.0)	0.460
Family function domain			
12. Had taken time off from work?	84 (45.9)	99 (54.1)	0.301
13. Had a financial impact on your family?	85 (46.4)	98 (53.6)	0.375
Overall ECOHIS			
Total ECOHIS score	1,349 (56.7)	1,029 (43.3)	**<0**.**001**

Statistically significant results (Chi-square test, *p* < 0.05) are highlighted in bold.

The mean total ECOHIS score was 18.4 (Table 3). No statistically significant differences were observed in mean total ECOHIS scores according to child sex across the six domains, either dimension, or the total ECOHIS score. In contrast, when stratified by age, children aged 5 years consistently showed higher mean scores than those aged 4 years. These differences were statistically significant in the “Self-image/social interaction”, “Parental distress”, and “Family function” domains, as well as in the “Family impact dimension” and the total ECOHIS score (*p* ≤ 0.041) (Table 3).

**Table 3:** Mean (SD) ECOHIS domain, ECOHIS dimension, and total ECOHIS score according to sex, age and total sample among preschool children.

Impacts	Sex			Age			Total sample
	Boys	Girls	*p*-value	4 years	5 years	*p*-value	
Oral symptoms domain	1.5 (1.5)	1.7 (1.6)	0.541	1.4 (1.5)	1.8 (1.5)	0.060	1.6 (1.6)
Functional limitations domain	5.0 (4.9)	5.4 (5.2)	0.585	4.5 (5.0)	5.9 (5.0)	0.064	5.2 (5.0)
Psychological impacts domain	2.7 (2.8)	2.5 (2.6)	0.818	2.3 (2.7)	2.9 (2.6)	0.062	2.6 (2.7)
Self-image/social interaction domain	2.1 (2.7)	1.9 (2.3)	0.855	1.6 (2.5)	2.3 (2.5)	**0**.**030**	2.0 (2.5)
Parental distress domain	3.4 (3.3)	3.5 (3.2)	0.770	2.9 (3.2)	4.0 (3.2)	**0**.**034**	3.5 (3.2)
Family function domain	3.5 (3.3)	3.6 (3.3)	0.867	3.0 (3.2)	4.0 (3.3)	**0**.**034**	3.5 (3.3)
Child impact dimension	11.3 (11.4)	11.5 (11.0)	0.829	9.8 (11.2)	13.0 (10.9)	0.056	11.4 (11.1)
Family impact dimension	6.9 (6.6)	7.1 (6.5)	0.837	6.0 (6.4)	8.0 (6.5)	**0**.**030**	7.0 (6.5)
Total ECOHIS score	18.2 (17.7)	18.6 (17.2)	0.853	15.8 (17.4)	20.9 (17.1)	**0**.**041**	18.4 (17.4)

Statistically significant results (Mann–Whitney *U*-test, *p* < 0.05) are highlighted in bold.

### 3.3 Associations among maternal age, dental caries experience, and children's OHRQoL

**Figure 1:**
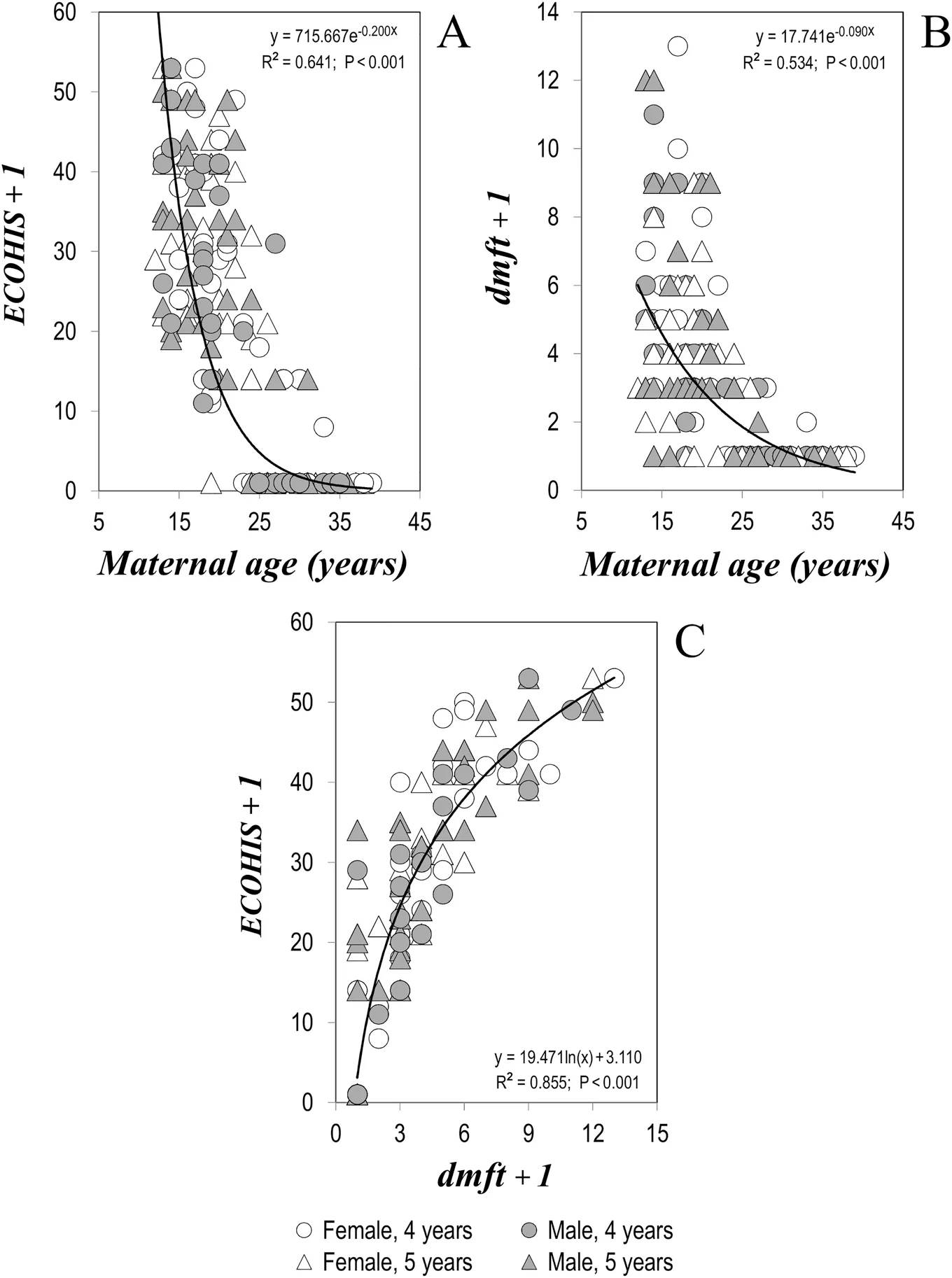
Relationships between maternal age, dental caries severity, and oral health–related quality of life (OHRQoL). **(A)** Association between maternal age and total ECOHIS score (ECOHIS+1). **(B)** Association between maternal age and dental caries experience (dmft+1). **(C)** Association between dental caries experience (dmft+1) and total ECOHIS score (ECOHIS+1). Points are color- and shape-coded by sex and age group. Points represent individual observations and lines indicate fitted non-linear regression models.

Maternal age was inversely associated with total ECOHIS score, with higher maternal age related with better oral health–related quality of life (*b* = −0.200, *p* < 0.001). A similar inverse association was observed between maternal age and dental caries experience, with maternal age significantly associated with a lower caries burden (*b* = −0.090, *p* < 0.001) (Figure 1). Exponential regression models indicated that each one-year increase in maternal age was associated with an average reduction of 18.1% in total ECOHIS scores and 8.6% in the dmft index. In turn, dental caries experience was positively associated with OHRQoL impairment, accounting for a large proportion of the variance (R^2^ = 85.5%, *p* < 0.001) (Figure 1). A doubling of caries burden was associated with an increase of approximately 13.5 points in the total ECOHIS score, with a steeper increase in OHRQoL impairment observed at lower levels of caries burden.

### 3.4 Mediation analysis

In the mediation analyses (Table 4), dental caries experience remained positively associated with worse OHRQoL after adjustment for maternal age (path *b*, on the natural log-transformed scale: *b* = 1.281, *p* < 0.001). After including ln(dmft+1) in the model, the direct association between maternal age and OHRQoL was attenuated but remained statistically significant (path *c*′: *b* = −0.084; *p* < 0.001). The indirect effect of maternal age on OHRQoL through dental caries experience was statistically significant (*a* × *b* = −0.116; *p* < 0.001). Overall, 57.8% of the total association between maternal age and OHRQoL was mediated by dental caries experience.

**Table 4:** Mediation analysis of the association between maternal age and oral health–related quality of life (OHRQoL), with dental caries experience (dmft) as mediator (natural log-transformed outcome scale).

Effect	Estimate	SE	*p*-value	95% CI
*a* (X → M)	−0.0902	0.0063	**<0**.**001**	
*b* (M → Y | X)	1.2813	0.0921	**<0**.**001**	
*c* (total effect: X → Y)	−0.2001	0.0111	**<0**.**001**	
*c*′ (direct effect: X → Y | M)	−0.0845	0.0114	**<0**.**001**	
Indirect effect (*a* × *b*)	−0.1156	0.0116		**−0.1503 to −0.0893**
Proportion mediated (%)	57.8			

X: maternal age; M: dental caries experience (dmft); Y: oral health–related quality of life (ECOHIS). Path *a* represents the association between maternal age and natural log-transformed dental caries experience; path *b*, the association between natural log-transformed dental caries experience and natural log-transformed ECOHIS score after adjustment for maternal age; path *c*, the association between maternal age and natural log-transformed ECOHIS score before accounting for dental caries experience; and path *c*′, the association between maternal age and natural log-transformed ECOHIS score after accounting for dental caries experience. The indirect effect (*a* × *b*) corresponds to the mediated effect of maternal age on children's OHRQoL through dental caries experience. The proportion mediated represents the percentage of the total effect explained by the indirect pathway. Statistically significant effects (*p* < 0.05 or 95% CI not including zero) are highlighted in bold.

## 4 Discussion

A high burden of oral health impacts was observed in this population, as reflected by the elevated mean total ECOHIS score (18.4). Although more than half of all ECOHIS item responses (56.7%) corresponded to the low-frequency impact categories (“never” or “hardly ever”), the overall score was considerably higher than those previously reported in studies conducted among children of similar age in several countries, including Brazil (49), China (50), Portugal (51), Sweden (43), and the United States (40), where 87.3%–94.9% of caregivers reported minimal impact and mean total ECOHIS scores ranged from 2.2 to 5.8. By contrast, the observed values were more comparable to those reported in Israel (52), Peru (53), and Turkey (42), where the proportion of positively connoted responses ranged from 62.6% to 68.3% and mean total ECOHIS scores ranged from 12.7 to 15.5. The observed score also considerably exceeded values previously reported in Chile, where mean total ECOHIS scores ranged from 2.6 to 4.5. Although some of these differences may be partially explained by variations in age composition across studies, particularly in studies including younger children, the magnitude of the observed score remained substantially higher. This finding is in keeping with the well-documented socioeconomic gradients observed in ECC and OHRQoL and suggests that preschool children living in the study area experience oral health challenges comparable to those reported in other socially vulnerable populations (2–5).

Within the Child impact dimension, the item “Had pain in the teeth, mouth, or jaws” showed the highest proportion of negatively connoted responses, reaching 50.8% of cases. In contrast, the remaining items predominantly showed positive response patterns, several of which were statistically significant. This pattern suggests that, despite the presence of dental caries, caregivers often perceive the impact of oral health on children's daily functioning as relatively limited, except when pain is involved. Conversely, in the Family impact dimension, all items displayed a predominance of negatively connoted responses, although none reached statistical significance. Together, these findings underscore the central role of pain in the relationship between oral disease and children's OHRQoL deterioration and may also reflect caregivers’ tendency to internalize responsibility for their children's oral suffering, even in the absence of severe oral health problems (54, 55).

Items related to avoiding smiling and avoiding speaking were among those with the lowest reported levels of impact. This pattern has been described in previous studies applying ECOHIS in preschool populations (47, 48, 50, 56) and aligns with child psychological development, as self-image perception and social comparison tend to consolidate around six years of age (57). Although no significant sex differences were found across domains—indicating that caregivers perceive oral health problems similarly in daughters and sons—a greater impact on OHRQoL was observed in the Self-image/social interaction domain in 5-year-old children. Additionally, higher impacts were identified in the Parental Distress and Family Function domains, as well as in the total ECOHIS score. These differences may indicate that by five years of age children already experience measurable social impacts on OHRQoL, but they may also reflect the greater severity and cumulative burden of oral health problems evident at that age (21, 58).

Dental caries experience accounted for a substantial proportion of the variability in the total ECOHIS score (R^2^ = 85.5%). This high explanatory power may reflect the strong conceptual, clinical, and empirical relationship between dental caries experience and oral health–related quality of life. As dental caries constitutes a major source of symptoms, functional limitations, and psychosocial impacts captured by the ECOHIS instrument, a strong association between these variables is biologically plausible. This finding should therefore be interpreted in the context of the close correspondence between the disease process and the outcome being measured, rather than as evidence of an unusually strong causal relationship. This interpretation is consistent with the broader body of evidence showing that reductions in ECC are associated with improvements in children's OHRQoL and family well-being (10–18).

In this study, caries diagnosis followed WHO criteria, which do not include non-cavitated lesions. This approach may underestimate the true disease burden and fail to capture early pathological changes with potential long-term consequences. However, previous studies have shown that non-cavitated lesions are generally not associated with measurable deterioration in OHRQoL, likely due to the absence of pain or evident aesthetic consequences (59). Accordingly, the fact that some children with dmft = 0 have elevated total ECOHIS scores suggests that other oral conditions not assessed in this study may influence OHRQoL even at early ages, such as dentomaxillary anomalies (54, 60).

Maternal age was inversely associated with both dental caries experience and children's OHRQoL, with children of younger mothers experiencing the greatest disease burden and quality of life impairment. However, these findings should not be interpreted solely as adverse outcomes associated with younger maternal age, but rather as reflecting a gradient across the maternal age continuum, suggesting that maternal age may capture broader life-course, social, and contextual conditions that shape children's oral health outcomes. This interpretation is also consistent with the educational trajectories observed among younger mothers in the study population, many of whom were still completing their education. Likewise, the lower disease burden observed among children of older mothers should instead be interpreted in the context of broader circumstances that may accompany later motherhood. These findings build upon previous studies that have generally reported weak inverse associations between maternal age and ECC or OHRQoL, many of which relied on dichotomized maternal age or limited age ranges (11, 34–38).

The mediation analysis suggested that dental caries represents an important pathway through which maternal age is associated with children's OHRQoL, accounting for 57.8% of the total association. These results suggest that dental caries is not merely correlated with children's OHRQoL but constitutes one of the principal pathways linking maternal age to this outcome in the study population. Specifically, increasing maternal age was associated with lower dental caries experience, whereas higher dental caries experience was associated with poorer OHRQoL. Accordingly, the negative indirect effect is consistent with a protective pathway linking increasing maternal age to better OHRQoL through reduced dental caries experience. Nevertheless, the persistence of a significant direct effect after accounting for dental caries indicates that additional pathways beyond caries are also likely to contribute to this association. The magnitude of the association between maternal age, dental caries, and OHRQoL was also reflected in the model-based estimates, which illustrated the marked gradient in both outcomes across the maternal age continuum observed in the study population. In this model, children of mothers aged 14 years would be expected to have a total ECOHIS score of 42.5, compared with 0.5 among children of mothers aged 31 years. A similar pattern was observed for dental caries experience, with mean dmft values ranging from 4.8 among children of mothers aged ≤14 years to 0.1 among those of mothers aged ≥31 years. Evidence further supports the relevance of maternal and caregiver-related characteristics, including educational attainment, oral health literacy, and caregiving behaviors, in shaping children's oral health outcomes and OHRQoL (61–65). These factors may also help explain the residual direct association between maternal age and children's OHRQoL observed after accounting for dental caries, reinforcing the interpretation of maternal age as a proxy for broader life-course determinants of child health (66, 67) that encompass underlying social and contextual conditions shaping children's oral health and well-being. Within this framework, the high overall burden of ECC and impaired children's OHRQoL observed in this population may largely reflect the disproportionate burden of disease concentrated among children of adolescent and younger mothers, whereas children of older mothers exhibited oral health profiles that more closely resembled those reported in socially advantaged populations. These findings highlight the coexistence of distinct risk profiles within the same population and suggest that the overall population burden may be strongly influenced by the subgroup of children bearing the greatest disease burden.

Within the Chilean context, the present results are particularly relevant. Although sustained public policies and preventive programs have contributed to a decline in ECC prevalence (19–21), the disease continues to disproportionately affect children from socioeconomically disadvantaged backgrounds (22–30). The high proportion of adolescent and very young mothers in the present study may therefore reflect the coexistence of structural conditions that contribute to the persistence of oral health inequalities from early childhood.

From a public health perspective, these findings support strengthening oral health strategies through a life-course and equity-based approach. Despite the availability of preventive programs and publicly funded dental services, the persistence of ECC and its impact on children's OHRQoL suggests that barriers extending beyond service availability—including delayed utilization, caregiver oral health literacy, and broader social determinants—remain important. Given the concentration of disease burden among children of adolescent and young mothers—whose pregnancies are often associated with social vulnerability (68)—preventive interventions integrated into maternal and child health services, particularly during pregnancy and early childhood, may be especially relevant. Complementing these strategies with community-based oral health education in primary healthcare facilities, day-care centres, and schools may further contribute to reducing oral health inequalities from the earliest stages of life.

### 4.1 Strengths and limitations

This study has several strengths. The regression and mediation analyses treated maternal age as a continuous variable, allowing a more comprehensive assessment of its association with children's oral health outcomes across the maternal age continuum. The mediation analysis further provided insight into the potential role of dental caries in the association between maternal age and children's OHRQoL. In addition, the high participation rate within the target population minimized the potential for selection bias within the study setting.

Several limitations should also be acknowledged. First, the cross-sectional design precludes establishing temporal relationships among maternal age, dental caries experience, and children's OHRQoL. Accordingly, the mediation analysis should be interpreted as describing patterns of association compatible with a mediational framework rather than evidence of causal mediation. Second, although all oral examinations were performed by a single examiner using standardized diagnostic criteria, no formal calibration or reproducibility assessment was conducted. While the use of a single examiner likely reduced inter-examiner variability, examiner reliability could not be formally quantified, and some degree of measurement error in dental caries assessment cannot be excluded. Third, part of the study data was obtained through caregiver interviews, which are inherently subject to recall and reporting bias, particularly regarding subjective aspects of children's oral health and quality of life. Fourth, although maternal age appeared to capture important social and contextual dimensions, residual confounding by unmeasured socioeconomic and behavioral factors, including household socioeconomic conditions, oral health practices, dietary habits, access to dental care, and other caregiver-related behaviors, cannot be excluded. Maternal educational level was not included as a covariate in the primary analytical models because it was measured at the time of interview rather than at childbirth and may therefore partially reflect life-course trajectories associated with early motherhood. Nevertheless, exploratory analyses incorporating maternal educational level yielded substantively similar association patterns. Finally, the study was conducted in a socioeconomically vulnerable urban population from a single public primary healthcare centre in northern Chile, which may limit the generalizability of these findings to populations with different demographic, socioeconomic, healthcare, and cultural contexts.

## 5 Conclusion

In the study population, the mediation analysis suggested that dental caries constituted an important component of the observed association between maternal age and children's oral health–related quality of life. Considering maternal age as a continuum may provide a more comprehensive understanding of inequalities in early childhood oral health. The observed associations are consistent with the interpretation of maternal age as a marker of broader social and contextual conditions influencing children's early oral health, rather than as an isolated demographic characteristic. Collectively, these findings support strengthening preventive oral health strategies through a life-course and equity-based approach, with particular emphasis on reducing oral health inequalities among socially vulnerable children and families.

## Data Availability

The datasets generated and analyzed during the current study are available from the corresponding author upon reasonable request for research purposes, subject to ethical approval and applicable institutional requirements.

## References

[1] Bernabe E Marcenes W Abdulkader RS Abreu LG Afzal S Alhalaiqa FN. Trends in the global, regional, and national burden of oral conditions from 1990 to 2021: a systematic analysis for the global burden of disease study 2021. Lancet. (2025) 405:897–910. 10.1016/S0140-6736(24)02811-3 40024264

[2] Maklennan A Borg-Bartolo R Wierichs RJ Esteves-Oliveira M Campus G. A systematic review and meta-analysis on early childhood caries global data. BMC Oral Health. (2024) 24:835. 10.1186/s12903-024-04605-y 39049051 PMC11267837

[3] Wen PYF Chen MX Zhong YJ Dong QQ Wong HM. Global burden and inequality of dental caries, 1990 to 2019. J Dent Res. (2022) 101:392–9. 10.1177/00220345211056247 34852668

[4] Uribe SE Innes N Maldupa I. The global prevalence of early childhood caries: a systematic review with meta-analysis using the WHO diagnostic criteria. Int J Paediatr Dent. (2021) 31:817–30. 10.1111/ipd.12783 33735529

[5] Foláyan Mẹ´ẹ´O De Barros Coelho EMR Feldens CA Gaffar B Virtanen JI Abodunrin OR. A scoping review on early childhood caries and inequalities using the sustainable development goal 10 framework. BMC Oral Health. (2025) 25:219. 10.1186/s12903-025-05587-1 39930428 PMC11812211

[6] Bernabe E Marcenes W Hernandez CR Bailey J Abreu LG Alipour V. Global, regional, and national levels and trends in burden of oral conditions from 1990 to 2017: a systematic analysis for the global burden of disease 2017 study. J Dent Res. (2020) 99:362–73. 10.1177/0022034520908533 32122215 PMC7088322

[7] Bencze Z Mahrouseh N Andrade CAS Kovács N Varga O. The burden of early childhood caries in children under 5 years old in the European union and associated risk factors: an ecological study. Nutrients. (2021) 13:455. 10.3390/nu13020455 33573027 PMC7911369

[8] Yousaf M Aslam T Saeed S Sarfraz A Sarfraz Z Cherrez-Ojeda I. Individual, family, and socioeconomic contributors to dental caries in children from low- and middle-income countries. Int J Environ Res Public Health. (2022) 19:7114. 10.3390/ijerph19127114 35742362 PMC9222700

[9] Shrestha SK Arora A Manohar N Ekanayake K Foster J. Association of breastfeeding and early childhood caries: a systematic review and meta-analysis. Nutrients. (2024) 16:1355. 10.3390/nu16091355 38732602 PMC11085424

[10] Abanto J Carvalho TS Mendes FM Wanderley MT Bönecker M Raggio DP. Impact of oral diseases and disorders on oral health-related quality of life of preschool children. Community Dent Oral Epidemiol. (2011) 39:105–14. 10.1111/j.1600-0528.2010.00580.x 21029148

[11] Martins-Júnior PA Vieira-Andrade RG Corrêa-Faria P Oliveira-Ferreira F Marques LS ML R-J. Impact of early childhood caries on the oral health-related quality of life of preschool children and their parents. Caries Res. (2013) 47:211–8. 10.1159/000345534 23257929

[12] Kramer PF Feldens CA Ferreira SH Bervian J Rodrigues PH Peres MA. Exploring the impact of oral diseases and disorders on quality of life of preschool children. Community Dent Oral Epidemiol. (2013) 41:327–35. 10.1111/cdoe.12035 23330729

[13] Ramos-Jorge J Alencar BM Pordeus IA Soares MEC Marques LS Ramos-Jorge ML. Impact of dental caries on quality of life among preschool children. Eur J Oral Sci. (2015) 123:88–95. 10.1111/eos.12166 25557987

[14] Zaror C Matamala-Santander A Ferrer M Rivera-Mendoza F Espinoza-Espinoza G Martínez-Zapata MJ. Impact of early childhood caries on oral health-related quality of life: a systematic review and meta-analysis. Int J Dent Hyg. (2022) 20:120–35. 10.1111/idh.12494 33825317

[15] Pakkhesal M Riyahi E Naghavi Alhosseini A Amdjadi P Behnampour N. Impact of dental caries on oral health-related quality of life among preschool children. BMC Oral Health. (2021) 21:68. 10.1186/s12903-021-01396-4 33588827 PMC7885600

[16] Yactayo-Alburquerque MT Alen-Méndez ML Azañedo D Comandé D Hernández-Vásquez A. Impact of oral diseases on oral health-related quality of life: a systematic review of studies conducted in Latin America and the Caribbean. PLoS One. (2021) 16:e0252578. 10.1371/journal.pone.0252578 34077473 PMC8171960

[17] Kurt A Bolat D Hatipoğlu Ö. Impact of the severity and extension of dental caries lesions on preschool children’s oral health-related quality of life. BMC Oral Health. (2025) 25:210. 10.1186/s12903-025-05549-7 39923039 PMC11807310

[18] Moghaddam LF Vettore MV Bayani A Bayat A-H Ahounbar E Hemmat M. Association of oral health status and socioeconomic determinants with oral health-related quality of life among children. BMC Pediatr. (2020) 20:489. 10.1186/s12887-020-02371-8 33092562 PMC7579886

[19] Danke K Carvajal C Borgeat M Carvajal P. Tendencia de niños y niñas de 6 años libres de caries en Chile entre los años 2012 y 2019. Int J Interdiscip Dent. (2022) 15:33–8. 10.4067/S2452-55882022000100033

[20] Cifuentes-Harris C Sánchez-Recio R. Políticas públicas odontológicas y su relación con historial de caries en menores controlados en atención pública de Chile. Int J Odontostomatol. (2022) 16:147–58. 10.4067/S0718-381X2022000100147

[21] Morales E Lancellotti D. Salud bucal de niños y niñas intervenidos por el programa odontológico Sembrando Sonrisas, Comuna de Ovalle, año 2019. Int J Interdiscip Dent. (2023) 16:40–4. 10.4067/S2452-55882023000100040

[22] Hoffmeister L Moya P Vidal C Benadof D. Factors associated with early childhood caries in Chile. Gac Sanit. (2016) 30:59–62. 10.1016/j.gaceta.2015.09.005 26655206

[23] Espinoza-Espinoza G Pineda P Atala-Acevedo C Muñoz-Millán P Muñoz S Weits A. Prevalencia y severidad de caries dental en los niños beneficiarios del programa de salud oral asociados a escuelas de Chile. Int J Odontostomatol. (2021) 15:166–74. 10.4067/S0718-381X2021000100166

[24] Echeverria-López S Henríquez-D’Aquino E Werlinger-Cruces F Villarroel-Díaz T Lanas-Soza M. Determinantes de caries temprana de la infancia en niños en riesgo social. Int J Interdiscip Dent. (2020) 13:26–9. 10.4067/S2452-55882020000100026

[25] Mariño RJ Onetto JE. Caries experience in urban and rural Chilean 3-year-olds. Community Dent Oral Epidemiol. (1995) 23:60–1. 10.1111/j.1600-0528.1995.tb00199.x 7774179

[26] Zaror-Sánchez C Pineda-Toledo P Orellana-Cáceres JJ. Prevalencia de caries temprana de la infancia y sus factores asociados en niños chilenos de 2 y 4 años. Int J Odontostomatol. (2011) 5:171–7. 10.4067/S0718-381X2011000200010

[27] Uribe S Rodriguez M Peigna G Provoste P Jara L. Prevalencia de caries temprana de la infancia en zona rural del sur de Chile, 2013. Ciencia Odontológica. (2020) 10:97–104.

[28] Giacaman RA Bustos IP Bravo-León V Mariño RJ. Impact of rurality on the oral health status of 6-year-old children from central Chile: the EpiMaule study. Rural Remote Health. (2015) 15:3135. 10.22605/RRH3135 26108477

[29] Espinoza-Espinoza G Muñoz-Millán P Vergara-González C Atala-Acevedo C Zaror C. Prevalence of early childhood caries in non-fluoridated rural areas of Chile. J Oral Res. (2016) 5:307–13. 10.17126/joralres.2016.064

[30] Monsalves MJ Espinoza I Moya P Aubert J Durán D Arteaga O. Structural determinants explain caries differences among preschool children in Chile’s metropolitan region. BMC Oral Health. (2023) 23:136. 10.1186/s12903-023-02778-6 36894931 PMC9996898

[31] Zaror C Atala-Acevedo C Espinoza-Espinoza G Muñoz-Millán P Muñoz S Martínez-Zapata MJ. Cross-cultural adaptation and psychometric evaluation of the early childhood oral health impact scale (ECOHIS) in Chilean population. Health Qual Life Outcomes. (2018) 16:232. 10.1186/s12955-018-1057-x 30554568 PMC6296046

[32] Núñez-Contreras J Hofer-Durán P Sinsay-Schmeisser J Zaror C. Impacto de las condiciones sociodemográficas y orales en la calidad de vida relacionada a la salud oral en preescolares de Temuco, Chile. Int J Odontostomatol. (2021) 15:503–12. 10.4067/S0718-381X2021000200503

[33] Cartes-Velásquez R Nauduam-Elgueta Y Sandoval-Bustos G Campos V León-Manco RA Luengo L. Factors associated with oral health-related quality of life in preschoolers of concepción, Chile: a cross-sectional study. Pesqui Bras Odontopediatria Clín Integr. (2022) 22:e210245. 10.1590/pboci.2022.074

[34] Mattila ML Rautava P Sillanpää M Paunio P. Caries in five-year-old children and associations with family-related factors. J Dent Res. (2000) 79:875–81. 10.1177/00220345000790031501 10765963

[35] Hallett KB O'Rourke PK. Social and behavioural determinants of early childhood caries. Aust Dent J. (2003) 48:27–33. 10.1111/j.1834-7819.2003.tb00005.x 14640154

[36] Pinto GDS Azevedo MS Goettems ML Correa MB Pinheiro RT Demarco FF. Are maternal factors predictors for early childhood caries? Results from a cohort in southern Brazil. Braz Dent J. (2017) 28:391–7. 10.1590/0103-6440201601047 29297562

[37] Yang L Zhao S Zhu Y Lai G Wang J. Oral health-related quality of life and associated factors among a sample from east China with severe early childhood caries: a cross-sectional study. BMC Oral Health. (2023) 23:837. 10.1186/s12903-023-03560-4 37936111 PMC10629075

[38] Palacios J Boonchieng W Poprom N Nirunsittirat A Luengo L. Correlation between caregiver’s oral health literacy and children’s oral health-related quality of life in rural Chile. Nat Life Sci Commun. (2026) 25:e2026022. 10.12982/NLSC.2026.022

[39] World Health Organization. Oral Health Surveys: Basic Methods. 5th ed. Geneva: World Health Organization (2013).

[40] Pahel BT Rozier RG Slade GD. Parental perceptions of children’s oral health: the early childhood oral health impact scale (ECOHIS). Health Qual Life Outcomes. (2007) 5:6. 10.1186/1477-7525-5-6 17263880 PMC1802739

[41] Zaror C Pardo Y Espinoza-Espinoza G Pont À Muñoz-Millán P Martínez-Zapata MJ. Assessing oral health-related quality of life in children and adolescents: a systematic review and standardized comparison of available instruments. Clin Oral Investig. (2019) 23:65–79. 10.1007/s00784-018-2406-1 29569021

[42] Peker K Uysal Ö Bermek G. Cross-cultural adaptation and preliminary validation of the Turkish version of the early childhood oral health impact scale among 5–6-year-old children. Health Qual Life Outcomes. (2011) 9:118. 10.1186/1477-7525-9-118 22192577 PMC3310831

[43] Sabel N Ylander LO Ståhlberg SE Robertson A. Dental caries and oral health-related quality of life in preschoolers: introducing the Swedish version of ECOHIS. Acta Odontol Scand. (2024) 83:47–53. 10.1080/00016357.2023.2287235 38032108 PMC11302645

[44] Osborne JW. Improving your data transformations: applying the box-cox transformation. Pract Assess Res Eval. (2010) 15:1–9. 10.7275/qbpc-gk17

[45] Baron RM Kenny DA. The moderator–mediator variable distinction in social psychological research: conceptual, strategic, and statistical considerations. J Pers Soc Psychol. (1986) 51:1173–82. 10.1037/0022-3514.51.6.1173 3806354

[46] Sobel ME. Asymptotic confidence intervals for indirect effects in structural equation models. In: Leinhardt S, editor. Sociological Methodology 1982. Washington, DC: American Sociological Association (1982). p. 290–312.

[47] R Core Team. R: A Language and Environment for Statistical Computing. Vienna: R Foundation for Statistical Computing (2024).

[48] Addinsoft. XLSTAT Statistical and Data Analysis Solution (Version 2016.1). Paris: Addinsoft (2016).

[49] Scarpelli AC Oliveira BH Tesch FC Leão AT Pordeus IA Paiva SM. Psychometric properties of the Brazilian version of the early childhood oral health impact scale (B-ECOHIS). BMC Oral Health. (2011) 11:19. 10.1186/1472-6831-11-19 21668959 PMC3125235

[50] Duangthip D Gao SS Chen KJ Lo ECM Chu CH. Oral health-related quality of life and caries experience of Hong Kong preschool children. Int Dent J. (2020) 70:100–7. 10.1111/idj.12526 31642058 PMC9379145

[51] Correia C Ribeiro Graça S Mendes S. Early childhood oral health impact scale: psychometric evaluation in Portuguese preschoolers. Acta Stomatol Croat. (2024) 58:39–51. 10.15644/asc58/1/4 38562224 PMC10981910

[52] Gaon O Natapov L Ram D Zusman SP Fux-Noy A. Validation of a hebrew version of the early childhood oral health impact scale. Front Oral Health. (2025) 6:1543327. 10.3389/froh.2025.1543327 40395723 PMC12089046

[53] López-Ramos RP García-Rupaya CR. Quality of life and oral problems in preschool children in huaura, Lima. Rev Estomatol Herediana. (2014) 23:139. 10.20453/reh.v23i3.24

[54] Abanto J Tello G Bonini GC Oliveira LB Murakami C Bönecker M. Impact of traumatic dental injuries and malocclusions on quality of life of preschool children: a population-based study. Int J Paediatr Dent. (2015) 25:18–28. 10.1111/ipd.12092 24387748

[55] Gomes MC Clementino MA Pinto-Sarmento TC Martins CC Granville-Garcia AF Paiva SM. Association between parental guilt and oral health problems in preschool children: a hierarchical approach. BMC Public Health. (2014) 14:854. 10.1186/1471-2458-14-854 25128429 PMC4150983

[56] Martins-Júnior PA Ramos-Jorge J Paiva SM Marques LS Ramos-Jorge ML. Validations of the Brazilian version of the early childhood oral health impact scale (ECOHIS). Cad Saude Publica. (2012) 28:367–74. 10.1590/S0102-311X2012000200015 22331162

[57] Harter S. The Construction of the Self: Developmental and Sociocultural Foundations. 2nd ed. New York: Guilford Press (2012).

[58] Dăguci L Bătăiosu M Andrei OC Scrieciu M Margaritescu C Dascălu I. Risk of dental caries for children aged 4 to 6 in craiova. Curr Health Sci J. (2016) 42:145–50. 10.12865/CHSJ.42.02.05 30568825 PMC6256160

[59] Guedes RS Ardenghi TM Piovesan C Emmanuelli B Mendes FM. Influence of initial caries lesions on quality of life in preschool children: a 2-year cohort study. Community Dent Oral Epidemiol. (2016) 44:292–300. 10.1111/cdoe.12217 26892250

[60] Kragt L Dhamo B Wolvius EB Ongkosuwito EM. The impact of malocclusions on oral health-related quality of life in children: a systematic review and meta-analysis. Clin Oral Investig. (2016) 20:1881–94. 10.1007/s00784-015-1681-3 26635095 PMC5069349

[61] Alzahrani AY El Meligy O Bahdila D Aljawi R Bamashmous NO Almushayt A. The influence of parental oral health literacy on children’s oral health: a scoping review. J Clin Pediatr Dent. (2024) 48:16–25. 10.22514/jocpd.2024.074 39087210

[62] Zhang B Zhou F Liu Z Gong A Li M. Association of parental oral health literacy, early childhood oral health behaviors, and caries severity with oral health-related quality of life among preschool children. BMC Oral Health. (2025) 25:1808. 10.1186/s12903-025-07159-9 41267046 PMC12636172

[63] Foláyan MO Zuñiga RAA Mohebbi SZ Khami MR. Association between early childhood caries and parental educational status among children in ile-ife, Nigeria. Front Oral Health. (2025) 6:1581589. 10.3389/froh.2025.1581589 41170204 PMC12568565

[64] Blanco-Victorio DJ López-Luján NA Bernaola-Silva W Vicuña-Huaqui LA Cacñahuaray-Palomino R Diaz-Campos JS. Sociodemographic and clinical factors associated with early childhood caries in Peruvian pre-schoolers. BMC Oral Health. (2025) 25:125. 10.1186/s12903-025-05506-4 39849474 PMC11760088

[65] Bal D Keskin G. Oral health status and early childhood caries in preterm and full-term children: a cross-sectional study. BMC Oral Health. (2026) 26:856. 10.1186/s12903-026-08439-8 42050483 PMC13173730

[66] Kato T Yorifuji T Yamakawa M Inoue S Doi H Eboshida A. Association of maternal age with child health: a Japanese longitudinal study. PLoS One. (2017) 12:e0172544. 10.1371/journal.pone.0172544 28234951 PMC5325269

[67] Ahmad M Sechi C Vismara L. Advanced maternal age: a scoping review about the psychological impact on mothers, infants, and their relationship. Behav Sci. (2024) 14:147. 10.3390/bs14030147 38540450 PMC10968301

[68] World Health Organization. Adolescent Pregnancy. Geneva: WHO (2024). Available online at: https://www.who.int/news-room/fact-sheets/detail/adolescent-pregnancy

