# Supplementary material for: Maternal age and oral health–related quality of life in preschool children: the mediating role of dental caries in a cross-sectional study
Source: Front Oral Health. 2026 Aug 20;7:1863687. doi: 10.3389/froh.2026.1863687 (PMC13538890; doi:10.3389/froh.2026.1863687)
Supplement: Supplementary file 1 [file Table1.docx]

**Supplementary Table S1. Formal comparison of slopes between groups using interaction models for ECOHIS and dental caries experience** **(dmft)**

Model 1. Association between maternal age and OHRQoL (ln[ECOHIS+1]) including interaction terms

| **Term** | **Coefficient** | **SE** | **t** | **p-value** |
| --- | --- | --- | --- | --- |
| Intercept | 6.451 | 0.538 | 11.998 | **<0.001** |
| Sex [Male] | 0.343 | 0.789 | 0.434 | 0.664 |
| Child age [5 years] | -0.106 | 0.732 | -0.144 | 0.886 |
| Sex × Child age | 0.036 | 1.075 | 0.033 | 0.973 |
| Maternal age | -0.191 | 0.022 | -8.778 | **<0.001** |
| Maternal age × Sex | -0.028 | 0.032 | -0.877 | 0.382 |
| Maternal age × Child age | 0.005 | 0.031 | 0.163 | 0.871 |
| Maternal age × Sex × Child age | 0.006 | 0.046 | 0.134 | 0.894 |

Model 2. Association between maternal age and dental caries experience (ln[dmft+1]) including interaction terms

| **Term** | **Coefficient** | **SE** | **t** | **p-value** |
| --- | --- | --- | --- | --- |
| Intercept | 3.007 | 0.304 | 9.906 | **<0.001** |
| Sex [Male] | 0.084 | 0.446 | 0.189 | 0.850 |
| Child age [5 years] | -0.334 | 0.413 | -0.809 | 0.420 |
| Sex × Child age | 0.061 | 0.607 | 0.101 | 0.920 |
| Maternal age | -0.093 | 0.012 | -7.604 | **<0.001** |
| Maternal age × Sex | -0.008 | 0.018 | -0.467 | 0.641 |
| Maternal age × Child age | 0.013 | 0.017 | 0.787 | 0.432 |
| Maternal age × Sex × Child age | 0.004 | 0.025 | 0.160 | 0.873 |

Model 3. Association between dental caries experience and OHRQoL (ECOHIS+1 vs ln[dmft+1]) including interaction terms

| **Term** | **Coefficient** | **SE** | **t** | **p-value** |
| --- | --- | --- | --- | --- |
| Intercept | 1.733 | 1.343 | 1.290 | 0.199 |
| Sex [Male] | 0.162 | 1.921 | 0.084 | 0.933 |
| Child age [5 years] | 2.582 | 1.982 | 1.303 | 0.194 |
| Sex × Child age | 0.451 | 2.824 | 0.160 | 0.873 |
| ln(dmft+1) | 20.469 | 1.191 | 17.186 | **<0.001** |
| ln(dmft+1) × Sex | -0.965 | 1.747 | -0.552 | 0.581 |
| ln(dmft+1) × Child age | -1.603 | 1.697 | -0.945 | 0.346 |
| ln(dmft+1) × Sex × Child age | 0.699 | 2.419 | 0.289 | 0.773 |

**Notes:**

- Reference categories: female sex and 4-year-old children.
- All models include main effects and corresponding interaction terms.
- Statistically significant associations (p < 0.05) are highlighted in bold.
